# Tetraspanins in Extracellular Vesicle Formation and Function

**DOI:** 10.3389/fimmu.2014.00442

**Published:** 2014-09-16

**Authors:** Zoraida Andreu, María Yáñez-Mó

**Affiliations:** ^1^Unidad de Investigación, Hospital Santa Cristina, Instituto de Investigación Sanitaria Princesa, Madrid, Spain

**Keywords:** biogenesis, extracellular vesicles, exosomes, biomarkers, tetraspanin-enriched microdomains, antigen presentation

## Abstract

Extracellular vesicles (EVs) represent a novel mechanism of intercellular communication as vehicles for intercellular transfer of functional membrane and cytosolic proteins, lipids, and RNAs. Microvesicles, ectosomes, shedding vesicles, microparticles, and exosomes are the most common terms to refer to the different kinds of EVs based on their origin, composition, size, and density. Exosomes have an endosomal origin and are released by many different cell types, participating in different physiological and/or pathological processes. Depending on their origin, they can alter the fate of recipient cells according to the information transferred. In the last two decades, EVs have become the focus of many studies because of their putative use as non-invasive biomarkers and their potential in bioengineering and clinical applications. In order to exploit this ability of EVs many aspects of their biology should be deciphered. Here, we review the mechanisms involved in EV biogenesis, assembly, recruitment of selected proteins, and genetic material as well as the uptake mechanisms by target cells in an effort to understand EV functions and their utility in clinical applications. In these contexts, the role of proteins from the tetraspanin superfamily, which are among the most abundant membrane proteins of EVs, will be highlighted.

## Introduction

Exosomes are extracellular vesicles (EVs) of 50–100 nm diameter released by many cell types when multivesicular bodies (MVBs) fuse with the plasma membrane at the end of the endocytic-recycling pathway (1). Different kinds of EVs can be isolated from all body fluids: blood plasma, serum, urine, saliva, breast milk, bronchial lavage fluid, amniotic fluid, cerebrospinal fluid, and malignant ascites (2) and have been envisioned as a novel mechanism of horizontal gene transfer. EVs contain a specific composition of lipids, mRNA, regulatory microRNAs, as well as proteins in a functionally active form (3). The transfer of this material can regulate gene expression and alter the fate of target cells (4), which may become activated, differentiated, or dedifferentiated according to the information received. As a result, EVs represent an important tool for intercellular communication and therefore play a key role in the regulation of physiological as well as pathological processes. EVs can induce endothelial cell activation, transfer metastatic capacity (5), or mediate the local spread of neurodegenerative diseases (6). On the other hand, EVs can mediate tissue repair and regeneration (7) and immune functions (8), so they have been proposed as ideal candidates for therapeutic applications.

Extracellular vesicles are highly enriched in tetraspanins, a protein superfamily that organize membrane microdomains termed tetraspanin-enriched microdomains (TEMs) by forming clusters and interacting with a large variety of transmembrane and cytosolic signaling proteins (9–11).

Among tetraspanins, CD9, CD63, CD81, CD82, and CD151 have a broad tissue distribution, while others are restricted to particular tissues, such as Tssc6, CD37, and CD53 in hematopoietic cells. Immunoelectron microscopy studies have showed that tetraspanins are abundant on various types of endocytic membranes (12) and have been widely used as exosomal markers. Because of their prevalence in EVs, we will review here the existing evidence that suggests a functional role for tetraspanins in the biogenesis, targeting and function of EVs.

## Tetraspanin Structure: The Key for Their Involvement in so Many Processes

Based on topological studies, tetraspanins have been defined as a superfamily of proteins with four transmembrane domains with some characteristic structural features. Despite their low sequence homology, tetraspanins contain four to six conserved extracellular cysteine residues, and polar residues within transmembrane domains. They also contain distinct palmitoylation sites and most members are also glycosylated (13).

Tetraspanins are involved in a multitude of biological processes that imply cell adhesion, motility, invasion, or membrane fusion as well as signaling and protein trafficking. Five critical regions of tetraspanins and their organization in membrane microdomains are fundamental for the role of tetraspanins in these biological processes (14, 15).

### Extracellular domains

Extracellular domains are the most variable regions in tetraspanins, being the least conserved between human and zebra fish. EC1 (first extracellular loop) also referred to as small extracellular loop (SEL), can be glycosylated in some tetraspanins and it is not recognized by monoclonal antibodies that recognize cell-surface epitopes. For that reason EC1 is thought not be involved in binding (13, 16). EC2 or LEL, the large extracellular loop of tetraspanins is better known based on structural studies of CD81–LEL (17, 18). This domain is divided into a constant region with conserved A, B, and E helices, suggested to mediate homodimerization through a hydrophobic surface, and a variable region with helices C and D flanking those sequences responsible for protein–protein interactions; although this overall structure of LEL has not been corroborated in other tetraspanins (13, 18). EC2 includes several conserved cysteine residues forming disulfide bonds, crucial for the correct EC2 folding (the CCG motif, one cysteine residue proximal to transmembrane four present in all tetraspanins, and the Pro–Xaa–Xaa–Cys (PXXC) motif in the majority, but not all, tetraspanins). Some tetraspanins present another pair of cysteine residues for a third disulfide bond (19, 20), while a small subgroup of closely related members contains eight Cys residues in the LEL (21, 22).

### Transmembrane domains

The high degree of conservation of tetraspanin-transmembrane stretches points to crucial functional roles. Mutations in TM domains have been associated with retinal disorders (23) and several studies have shown that conserved polar residues in TM domains 1, 3, and 4 can be responsible of the correct packing of TM domains (24, 25). They are responsible for a proper tetraspanin biosynthesis and maturation via intra-molecular interactions and contribute to the formation of TEMs through hydrophobic interactions between tetraspanins (26).

### Cytoplasmic domains

The high degree of inter-species conservation of the C-terminal domain could point to this region as crucial in defining the functional specificity for each tetraspanin. The C-terminal region usually presents crucial motifs involved in the sorting and targeting of tetraspanins to a determined intracellular location. The Gly-Tyr-Glu-Val-Met (GYEVM) sequence targets tetraspanin CD63 to the late endosomal–lysosomal compartment, while the motif YXXØ (Tyr-Xaa-Xaa-Ø, where Ø represents of an amino acid with a bulky hydrophobic side chain) is a sorting signal for clathrin-coated vesicles (27, 28). Potential tyrosine-based sorting sequences YXXØ are present in other 12 tetraspanins (12). Remarkably, in the absence of these motifs, their function could be replaced by interactions with tetraspanins that do contain targeting motifs, providing a molecular explanation for the wide distribution of tetraspanins in endosomes, late endosomes, lysosomes as well as the enrichment of tetraspanins in EVs (29).

The C-terminal domain of CD63 interacts with several subunits of adaptor protein (AP) complexes, linking the traffic of this tetraspanin to clathrin-dependent pathways (27). Among intracellular interacting proteins, CD63 was shown to directly bind to syntenin-1, a double PDZ domain-containing protein (30). Remarkably, a major role in exosome biogenesis was recently reported for syntenin-1 (31).

In addition, the C-terminal domain of tetraspanins can mediate interactions with cytoskeletal or signaling proteins. The interaction of tetraspanins with the cytoskeleton may occur via proteins of the ezrin–radixin–moesin (ERM) family, which in turn bind to actin (32). Tetraspanins CD9 and CD81, but not CD151, co-immunoprecipitate with ERM proteins (33). Immunoglobulin superfamily (IgSF) proteins such as EWI-2, EWI-F, ICAM-1, or VCAM-1, which are direct partners of tetraspanins CD9 and CD81, also present binding sites for actin-linking ERM proteins (33, 34). A proteomic study in human primary lymphoblasts and their derived EVs identified the association of CD81 with a large number of interacting partners, including alpha-actinin (35, 36). In the activation and adhesion of T cells, an inducible association of CD82 with the cytoskeletal matrix has been observed, so that CD82 induces spreading and development of membrane extensions, involving actin polymerization and contributing to T cell activation via their cytoplasmic domain (37).

Regarding the interaction of tetraspanins with signaling molecules, early mapping studies with chimeric tetraspanins suggested that N-terminal and C-terminal regions contained sites to recruit PKC and other signaling proteins (38, 39). CD9 and CD81 have been shown to associate to G proteins and the inclusion of GPCR 56 in TEM was described and supported by the identification of several protein G subunits (40, 41). The direct interaction of CD81 C-terminal domain with the small GTPase Rac (35) regulates tumor (35) and dendritic cell (DC) (42) migration.

### Palmitoylation sites

Palmitoylation, one of the major post-translational modifications that tetraspanins are subjected to, involves the covalent attachment of palmitate to juxtamembrane cysteine residues resulting in the acylation of the protein. For several proteins, palmitoylation is required for localization into detergent resistant membrane microdomains (43). Regarding tetraspanins and partner proteins, palmitoylation contributes to organization of tetraspanin–tetraspanin interactions, the basis for the formation of TEMs (44–46).

### Tetraspanin web, a functional membrane microdomain

**Figure 1:**
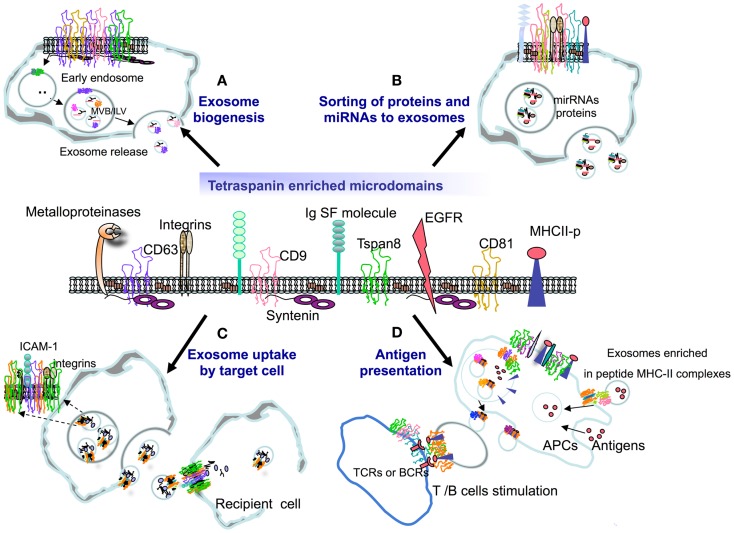
**Tetraspanins have the capacity to interact with several receptor and signaling molecules at the membrane, organizing specialized tetraspanin-enriched microdomains (TEMs) that may play a role in (A) EV biogenesis, (B) the selection of exosome cargo (proteins and miRNAs), (C) the binding and uptake of exosomes by target cells, or (D) the ability of exosomes to present antigen in the context of an immune response**.

Tetraspanins are able to concentrate on the plasma membrane and establish a set of interactions between themselves and with a variety of transmembrane and cytosolic proteins (Figure 1). Tetraspanins also associate with cholesterol (47) and gangliosides (48) forming specialized membrane platforms termed TEMs. Based on their biochemical properties and protein composition, TEMs represent a type of functional membrane microdomain different to other microdomains such as lipid rafts or caveolae (9, 11).

The main tetraspanin protein partners are integrins and IgSF members of adhesion receptors, signaling receptors, and enzymes such as metalloproteinases (11, 49). The interactions between tetraspanins and their protein partners are classified in three levels, based on their resistance to detergent disruption. Direct interactions with some integrins and IgSF partners are considered as type I interactions. Type II interactions include the majority of tetraspanin–tetraspanin interactions, which are stabilized by their palmitoylation. Weak, type III interactions, also stabilized by palmitoylation, occur with secondary partners (10).

This capacity of tetraspanins to interact laterally with membrane molecules and organize supramolecular complexes in cell membranes provides a molecular basis for their ability to modulate a wide range of fundamental biological and pathological processes (10, 11). In addition, given that tetraspanins and their associated proteins are abundant in EVs, the tetraspanin web could be key to understand how genetic information such as mRNA and microRNA, as well as functional proteins are selected to cargo EVs and transferred to target cells.

## Tetraspanins as Exosome Markers

Besides apoptotic bodies (ABs), healthy cells from different systems are able to secrete several types of membrane vesicles of endosomal and plasma membrane origin into the extracellular space. Because of the lack of a selective isolation procedure, and the high heterogeneity of EVs, there is not a definitive nomenclature for these vesicles. They have been broadly categorized based on their size and biogenesis into three main groups: i) microvesicles (MVs), also termed ectosomes, shedding vesicles, or microparticles. They are directly formed by outward budding from the plasma membrane and represent a very heterogeneous population with a size ranging from 50 to 1,000 nm diameter, ii) exosomes, with a size between 30 and 100 nm, formed as intraluminal vesicles (ILVs) in intracellular endosomal MVBs and released when MVBs fuse with the plasma membrane, and iii) ABs, with a size bigger than 1 μm and originated from apoptotic cells (3, 50).

Microvesicles and exosomes are both commonly found in extracellular fluids and may be produced by the same cell type (51, 52). Moreover, although some vesicles differ clearly from exosomes by their larger size, others are more difficult to separate since vesicles with a similar size can also bud at the plasma membrane (53). In addition, exosomes themselves represent a heterogeneous population given that MBV in some cell types contain ILVs of heterogeneous size and composition. Thus, features such as size and/or density cannot be used as strict criteria to define exosomes. Many isolation procedures and commercially available kits, in addition to other biochemical and imaging techniques such as immunoblotting, mass spectrometry, electron microscopy, flow cytometry, or nanoparticle tracking, should be considered cautiously because often they are not efficient in discriminating among differently sized EVs and/or membrane-free macromolecular aggregates. Research on optimization of these methodologies is currently a very active issue.

Because of these methodological issues, the identification of a selective subset of proteins in exosomes would be a valuable tool to identify and assess the purity of this kind of vesicles. Proteomic and lipidomic analyses show that exosomes have a defined lipid and protein composition. According to most recent proteomic results gathered in the ExoCarta and EVPedia databases (54, 55), exosomes have a defined protein signature, comprising conserved as well as cell type specific sets of exosomal proteins. In this context, exosomes have been described as highly enriched in tetraspanins (from 7- to 124-fold compared to their content in the parental cells) and tetraspanins have been proposed as possible exosome markers. Tetraspanins CD9, CD63, CD37, CD81, or CD82 are specially enriched in the membrane of exosomes and they are often used as exosome biomarkers. CD9 was first identified in exosomes from DCs (56). Several studies describe to CD63 and CD81 as the most frequently identified proteins in exosomes and are considered classical markers of exosomes. In fact, in many cells the bulk of CD63 has been described as typical in intracellular compartments of endosome/lysosomal origin (57). Proteomic analyses have identified tetraspanins CD63, CD81, CD82, CD53, and CD37 in B cell-derived exosomes being enriched >100-fold relative to transferrin receptor (1, 29).

However, this aspect has to be taken with caution since many tetraspanins are widely distributed in the plasma membrane, so that they may be present in other subpopulations of vesicles. Studies aimed to distinguish subpopulations of EVs from different cell types based on the presence of several tetraspanins have shown that in some cases this criterion on its own does not permit successful discrimination of exosomes from other EVs. Several studies have shown that classical markers of exosomes, such as CD63 and CD81, are enriched in vesicles with features of exosomes but which originate through budding from the plasma membrane and could not be distinguished from exosomes (53, 58, 59). CD9 was also found in large vesicles can thus not be considered as specific components of endosome-derived vesicles. (60). Most recently, CD81 and CD63 have been detected by flow cytometry in both MVs and exosomes secreted by three different cell lines (61).

Exosomes themselves comprise a heterogeneous vesicle population, also regarding tetraspanin expression. In this regard, *Rab27a* GTPase has been demonstrated to modulate exosome secretion (62) and the vesicular secretion of some conventional markers of exosomes such as the tetraspanin CD63, but does not affect the secretion of tetraspanin CD9. Taking advantage of Rab27a inhibition, the existence of at least two distinct populations of vesicles secreted by mouse mammary adenocarcinoma was demonstrated, and the presence of CD63 and CD9 was detected in both (60). Flotation onto sucrose gradients showed different proportions of CD63 and CD9, not only in fractions of densities classically described for exosomes, but also in non-exosomal density fractions, indicating the presence of an heterogeneous vesicle population (60). Finally, B cell-derived exosomes were shown to contain the tetraspanin markers CD9 and CD81, while CD63 was absent from these exosomes (63).

Thus, the identification and validation of tetraspanins as markers of exosomes or other vesicular types still deserves further standardization of isolation protocols as well as the implementation of single-vesicle immune-staining analyses. However, in any case, tetraspanin-based tools may represent powerful approaches in terms of EV enrichment for biomarker discovery and therapeutic use.

## Tetraspanins in EV Biogenesis

As mentioned before, exosomes originate as ILVs in late endosomes also termed MVBs (Figure 1A). The fate of MVBs may be the fusion with lysomes for degradation, when the proteins they contain have been selected through ubiquitination. Alternatively, some MVBs may fuse with the plasma membrane of the cell releasing ILVs as exosomes (1, 8). Given that exosomes derive from MVBs, mechanisms involved in MVBs and ILVs biogenesis are shared with exosomes, although the cellular machinery involved in their release is likely different.

The endosomal sorting complex required for transport (ESCRT) is composed by around 20 proteins assembled into 4 complexes (ESCRT-0, -I, -II, and -III) together with associated proteins (VPS4, VTA1, and ALIX) and is conserved from yeast to mammals (64). Both ESCRT-dependent and -independent mechanisms have been shown to be involved in the trafficking of proteins to exosomes and in their biogenesis but none of them are completely understood. The ESCRT-0 complex recognizes and sequesters ubiquitinated proteins in the endosomal membrane, ESCRT-I and -II complexes are responsible for membrane deformation into buds with sequestered cargo, while ESCRT-III drives vesicle scission. Distinct members of the ESCRT machinery have been involved in exosome biogenesis/secretion in different cell types (65). AlP1/Alix/Vps31, Tsg 101/Vps23, and ubiquitinated proteins are necessary for the secretion of exosomes by DCs (66, 67). The ESCRT-0 component Hrs is also required for exosome formation and/or secretion by DC, impacting on their antigen-presenting capacity (68). In reticulocytes, the transferrin receptor is generally fated for exosome secretion by interaction with Alix (69) and more recently, Alix has been described to be involved in exosome biogenesis and exosomal sorting of syndecans through its interaction with syntenin (31). A recent study using RNA interference (RNAi) to target 23 different components of the ESCRT machinery and associated proteins, highlights the cell-specificity of the mechanism of protein sorting and secretion in exosomes (65). The tumor suppressor protein p53 and its transcriptional target TSAP6 have been implicated in the regulation of exosome secretion (70), linked to the ESCRT-III component Chmp1A (71), illustrating potential couplings between signaling and exosome biogenesis.

Interestingly, in the absence of ESCRTs, MVBs, and exosomes may be formed, so that cells in which the four subunits of the ESCRT complex have been depleted are able to generate CD63-positive EVs (72). In antigen-presenting cells (APCs), the recruitment of MHC-II to exosomes is independent of MHC-II ubiquitination but in contrast, depends on the incorporation into CD9-enriched microdomains (73). In oligodendroglial cell lines, exosome biogenesis and secretion is dependent on the formation of ceramide by the shyngomyelinase enzyme, but does not require ESCRT function (74). Studies focused on melanogenesis have shown that mammalian cells have pathways for MVB formation independently of both ESCRTs and ceramide (75).

In this ESCRT-independent pathway, tetraspanins seem to play a fundamental role (73, 75). Regarding data based on tetraspanin-deficient mice, exosome secretion is defective in bone marrow dendritic cells (BMDCs) from CD9 knockout mice in comparison with their wild-type counterparts (76). In contrast, the absence of CD81 in lymphocytes does not affect exosome release (35). Others studies have shown the essential role of tetraspanin CD63 in the biogenesis of lysosome-related organelles (75), although exosome secretion was not directly assessed. shRNA knockdown of the tetraspanin protein CD63 in a B lymphoblastoid APC line led to increased CD4 T cell activation because of a significantly increased MHC-II-bearing exosome production (77).

The mechanisms of MV biogenesis, based on membrane blebbing, have been much less studied. External or internal stimuli promote calcium fluxes, regional changes in plasma membrane asymmetry leading to phosphatidylserine exposition, which in turn leads to modifications in membrane-cytoskeleton contacts, followed by membrane curvature, and vesicle scission (78). Actin–myosin interactions allow the contraction of cytoskeleton ending the budding process. These molecular events may be similar to those elicited by budding viruses. Tetraspanins in the plasma membrane are also forming specialized microdomains and their presence in shedding vesicles (61) has been reported. They have also been reported to conform specialized membrane regions for viral budding (79). Tetraspanins may induce membrane-curved structures (80), as demonstrated for a specialized tetraspanin, peripherin/RDS, in the retina (81). In addition, tetraspanin connections to the cytoskeleton may influence the fission process of the vesicles. However, all these hypotheses will have to wait for experimental evidence to be confirmed.

## Tetraspanins in EV Cargo Selection

### Intracellular routing of tetraspanin partners

To be directed to exosomes, membrane molecules have first to be exposed on the plasma membrane and internalized to the endosome compartments. Tetraspanins are involved in recycling routes between plasma membrane and several cellular organelles (Figure 1B) and regulate biosynthetic maturation and trafficking of their associated partners. One of the most dramatic examples is that of the dependence of CD19 expression on tetraspanin CD81 (82, 83). CD81 is involved in the proper maturation and trafficking of CD19 from the ER to the Golgi and to the cellular plasma membrane, where it takes part in the B cell co-receptor signaling complex formed by CD19–CD21–CD81. A reduced expression of CD19 in B cells from CD81−/− mice promotes an incorrect transition from pre-BII to the immature B stage, a phenotype that could not be rescued by CD9, the tetraspanin with the closest homology to CD81 (82, 83).

Tetraspanin CD82 associates with the alpha6 integrin, the epidermal growth factor receptor (EGFR) and the IgSF protein EWI-2. The co-internalization of CD82 with these partners promotes alterations in laminin adhesion and migration (84). CD82/KAI1 acts as a tumor suppressor modulating the activities of EGFR (85). Tetraspanin CD82/KAI1 has been described to suppress ligand-induced ubiquitination of EGFR after ligand binding, altering the rate of recruitment of the activated receptor to endosomes. Deletion of the C-terminal cytoplasmic domain of CD82 inhibits endocytic trafficking of the tetraspanin and compromises CD82 modulatory role on the endocytic trafficking of EGF receptor (84, 86).

Tetraspanins also control the trafficking of integrin complexes (87). The assembly of the complex between CD151 and alpha3beta1 integrin takes place early during the biosynthesis of the integrin heterodimer (88). The palmitoylation-deficient mutant of CD151 exhibited a decreased half-life (45), and the expression of this mutant in fibroblasts diminished the stability of the alpha3beta1 at the plasma membrane.

Somehow surprisingly, different tetraspanin/partner complexes may present different internalization rates on the same cells. Thus, evaluation of tetraspanin/integrin colocalization at the cell membrane or after PMA-induced internalization, showed that integrin alpha3 colocalization with CD9 and Tspan8 remains unaltered after internalization. In contrast, integrin alpha4 weakly colocalizes with CD9 and Tspan8 at the cell membrane but does so only with Tspan8 after internalization. Activation-induced Tspan8-internalization proceeds more rapidly than CD9 internalization and is accompanied by disassembly of the Tspan8-CD9–CD151 membrane complex in resting cells (89). Thus, different integrin/tetraspanin complexes were biochemically detected in lysates from cells or EVs. These results point to a rearrangement of the TEMs during internalization.

### Protein sorting to EVs

Consistent with the role of TEM in modulating internalization and recycling, different tetraspanin members have been shown to regulate protein sorting into EVs. Tetraspanins CD82 and CD9, by their association with E-cadherin and β-catenin, are necessary for the cellular export of β-catenin via EVs, thus modulating the wnt-signaling pathway (76). In mouse models of breast cancer, cancer-associated fibroblast-derived exosomes are enriched in tetraspanins CD63, CD81, and CD82 but only CD81 is responsible of Wnt 11 cargo to EVs. These EVs released into the tumor stroma are internalized by breast cancer cells, in which Wnt11 contributes to cell migration and metastasis (90). The melanosomal protein PMLE (amyloidogenic pigment cell-specific type I integral membrane protein) is sorted into ILVs by the tetraspanin CD63 in an ESCRTs-independent way (75). This sorting event is important to generate melanosome precursors so that the deletion of CD63 impairs amyloidogenesis and downstream melanosome morphogenesis (75). In contrast targeting of CD9P-1 to EVs occurs, at least partially, after silencing of its direct tetraspanin partners CD9 and CD81 (91).

Another set of molecules that may be targeted to EVs by insertion into TEM are metalloproteinases. The CD10 metalloproteinase, involved in the maturation of pre-B cells and migration of B cells to the blood circulation, has been shown to selectively associate with tetraspanin CD9. CD10 release in EVs was increased fivefold by stable expression of wild-type CD9 but not a chimeric CD9 containing the cytoplasmic C-terminal domain from CD82. Knockdown of CD9 expression promoted a twofold reduction in the amount of endogenous CD10 released with EVs. The release of CD10 peptidase activity on EVs in bone marrow may effectively regulate the extracellular matrix microenvironment (92). Tetraspanins also interact in membrane microdomains with the metalloproteinases ADAM10 and ADAM17 and control their sheddase activity (21, 22, 49, 93, 94). CD9 is associated with ADAM17 through its LEL domain on the surface of leukocytes and endothelial cells and regulates negatively the activity of ADAM17 (94). ADAM10 and ADAM17 have been shown to be also present in EVs in a functionally active form. Their sheddase activity on their substrates could be initiated in endosomal compartments and the product of their activity released as soluble molecules in EVs. For example, the cytokine TNF-α, or L1 and CD44 adhesion molecules, are cleaved by ADMA10 and ADAM17 and released in EVs by ovarian cancer or melanoma cells (95–97). The presence of ADAM17 and ADAM10 active forms in vesicles has been also detected in melanoma and HIV-1 infected T cells (98).

In the immune system, the sorting of MHC-I and MHC-II to exosomes seems to be dependent on their recruitment to TEMs (29, 73). Early proteomic and biochemical studies on B-, T-, and dendritic-cells-derived EVs evidence that EVs derived from these cells are enriched in tetraspanins CD9, CD63, CD81, CD82, MHC-I, and MHC-II (29, 56, 99). Characterization of physical interactions between proteins in detergent resistant membranes supported that the trafficking of MHC-II to B cell-derived EVs is dependent on their recruitment to membrane microdomains formed by tetraspanins CD37, CD53, CD63, CD81, and CD82 (100). In DCs, peptide-loaded MHC-II follows a different sorting route depending on the maturation state of the DCs (73). In immature DCs, peptide-loaded MHC-II is ubiquitinated for lysosomal degradation, but in mature DCs peptide-loaded MHC-II is driven by CD9 tetraspanin to MVBs, subsequently released as EVs and finally able to activate T cells (73). The presence of MHC-I and MHC-II associated to CD9 and CD81 has been also confirmed in B cells and B cells derived EVs (63). Interestingly, some MHC haplotypes were significantly underrepresented in EVs from CD81 deficient T-lymphoblasts (101). In contrast, silencing of CD63 tetraspanin does not affect the trafficking of MHC-II complexes to exosomes in DCs, although increases their secretion (77).

The role of tetraspanins in EV cargo selection (Figure 1B) has been corroborated in high throughput analyses. Comparison of EVs derived from a highly metastatic rat pancreatic adenocarcinoma cell line expressing Tspan8 with its wild-type counterpart suggests that Tspan8 contributes to a selective recruitment of proteins into EVs, including VCAM-1 and the integrin alpha4, which were involved in EV–EC binding and internalization (102). The genetic deletion of CD81 in primary mouse lymphoblasts impairs the inclusion in EVs of a selective repertoire of transmembrane CD81 partners, including MHC molecules, the B cell receptor, ICAM-1, and Rac (101). In this study, high throughput quantitative proteomics demonstrated that TEM interactions network accounts for 45% exosomal proteome, so that depletion of a given tetraspanin diminishes the concentration in EVs of some of their associated partners in the network (101). All these evidences suggest TEMs play a role in defining the protein content of EVs so that the proteome of TEM and that of exosomes are closely overlapping. However, since internalization rates of different TEM components are different and the ratio of plasma membrane versus endosome expression is different for different tetraspanins, the composition of TEM varies in different intracellular compartments. A more profound analysis of the dynamics of TEM-driven intermolecular interactions along the endocytic pathway is thus needed to fully understand how these specialized membrane platforms drive their components toward EVs.

### RNA sorting to EVs

Exosomes also contain a selected composition in small RNAs (vaultRNA, tRNAs, and miRNAs) (103) and specialized mechanisms are involved in their recruitment and loading to EVs (104). Several miRNAs present specific EXOmotifs (GGAG) that are able to bind to heterogeneous ribonucleoprotein A2B1 (hnRNPA2B1), which is responsible for the trafficking of these miRNAs into EVs (105). Other sequences and proteins have been described to regulate the loading of small RNAs to EVs (106, 107) Intriguingly, among the intracellular TEM interactome in human lymphoblasts many RNA-binding proteins were recovered (101). This connection could also provide a molecular mechanism for the recruitment of mRNA or miRNA into exosomes in which tetraspanins may be involved. Accordingly, in EV-derived from the metastatic rat pancreatic adenocarcinoma line expressing Tspan8 mentioned above, from 1,500 transcripts; 285 were enriched by >3-fold in Tspan8–EVs compared to EVs from cells not expressing Tspan8 (102).

Therefore, the TEMs act as specialized scaffolds for the compartmentalization of receptors and signaling proteins from the plasma membrane into EVs, playing a role in the sorting and selective recruitment of several proteins and possibly RNA. This scaffold would represent a potential target in the design of genetic therapies to route a desired agent to these natural nanocarriers.

## Tetraspanins in EV Targeting and Uptake

Although in some scenarios EVs may serve as extrusion moieties, with a functional role at the parental cell, the most spread function of EVs is their capacity to be selectively taken up by cells distal from the site of their release. This uptake may regulate gene expression or initiate the activation of signaling cascades in the recipient cells because of the EV molecular cargo (3, 108). Specificity in the uptake process of EVs by the target cells is another interesting point of EV biology to explore. For example, tumor-derived EVs play a significant role in the communication and interaction between the tumor and immune cells by suppressing the anti-tumoral immune response (108). But in addition, cancer cells-derived EVs transfer cancer-promoting factors to neighbor cells within the tumor microenviroment or to the circulation promoting cancer spread. Therefore, specific cell targeting may determine the functional outcome for EVs.

The targeting and uptake of exosomes by recipient cells is poorly understood. Adhesion molecules such as integrins and ICAMs, which are commonly inserted in TEMs (34, 109), are involved in the binding of exosomes to the target cell (Figure 1C). Exosome uptake by immune cells is mediated by ICAM-1/LFA1 (110–112). A membranous form of ICAM-1 found on tumor-derived exosomes, is able to bind to leukocytes and impair their adhesion to activated endothelial cells (113).

The tetraspanin-mediated sorting of specific adhesion receptors to EVs may influence the targeting of these EVs since several adhesion receptors and proteases depend on TEM for their sorting to EVs. However, to date, only a few studies have directly addressed the role of TEM in EV targeting and uptake (89, 114). Thus, as mentioned before, the expression of Tspan8 in rat adenocarcinoma increases the sorting of VCAM-1 and integrin alpha4 to EVs, eliciting the preferential binding of these EVs to endothelial cells (89, 102). In this context, ICAM-1 presented on EV surface seems to be functionally important for binding (114). In contrast, EVs derived from the highly metastatic rat pancreatic adenocarcinoma line BSp73ASML, which are enriched, in α6β4 and tetraspanins CD151 and Tspan8, preferentially target lung and lymph node stroma cells (114).

Regarding EV uptake, phagocytic processes dependent on dynamin2 and PI3K seem to be the dominant mode of EV uptake into recipient cells (115). However, depending on the recipient cell type, clathrin–dynamin–caveolae-dependent endocytosis and pinocytosis mechanisms may also operate (116, 117). In principle, the fusion of exosomes with the plasma membrane of recipient cells it is difficult at a neutral pH (118). In some scenarios, TEMs represent an alternative route of endocytosis, as reported for the entry of some viruses (119). Moreover, TEM regulates several fusion-dependent processes, ranging from sperm-egg fusion, to myoblast formation or viral-induced syncytia (19). The relevance of TEMs in exosome internalization or fusion deserves further analyses, since a better understanding of the role of TEM in EV-target cell binding and uptake is essential to improve the selectivity and specificity of EV message transfer under physiological and pathological conditions and for the optimization of modified EVs as selective therapeutic moieties.

## Tetraspanins and Antigen Presentation by EVs

### Antigen presentation by EVs

Early studies showed that immune cells such as T lymphocytes, B cells, and DCs are able to release EVs, and those EVs secreted by APCs were also able to present MHC–peptide complexes to specific T cells inducing an adaptive immune response (120–122). Several analyses addressing T cell activation have shown that EV-borne MHC–peptide complexes can directly bind to their cognate T cell receptor and activate primed CD4^+^ and CD8^+^ T cells (111, 123, 124) (Figure 1D). In addition, EVs captured by DCs also represent a source of antigens from the cells they derive (Figure 1D). EV-borne proteins are processed by DCs into peptides that associate to MHC molecules for eventual presentation to T lymphocytes (125–127).

The physiological state of the cells that secrete EVs is important for the intensity of T cell activation by EVs. EVs derived from mature DCs induce a better T cell activation than EVs derived from immature DCs and *in vivo* promote effector T cells and antibody responses (112, 123, 128, 129). EVs from mature DCs loaded with tumor antigen can directly induce anti-tumor immune responses of CD8^+^ T cells and activate naïve CD4^+^ T cells. In contrast, EVs derived from immature DCs need to be processed by APCs to induce an efficient T cell activation (130).

In some environments, EVs may also contain immunosuppressive molecules being able to inhibit immune cells and to promote tolerance. For instance, EVs delivered by intestinal epithelial cells have the ability to induce antigen-specific tolerance (131). Tolerance-inducing effects of EVs have been also described in transplant acceptance (132). Placenta, semen, milk, colostrum, and bronchoalveolar fluids are other environments where the presence of EVs containing immunosuppressive molecules has been demonstrated (133–136). Tumor-derived EVs may have either activating or inhibitory effects. EVs secreted by tumor cells have the ability to stimulate and initiate an anti-tumor-specific immune response by transferring tumor-specific antigens to DCs or other APCs (137). In contrast, tumor-derived EVs inhibit NK cell cytotoxic activity, DC differentiation from myeloid precursors, and T cell activation resulting in a decrease in T cell proliferation, cytotoxic activity, and finally increased tumor spread (121, 138–141).

Extracellular vesicles do not only contain proteins or processed peptides that can function as antigens but also transport miRNAs. In fact, EVs from T, B, and dendritic immune cells contain a different miRNA profile than that of their parental cells (142, 143). In addition, linked to the formation of the immune synapse (IS), during cognate immune interactions, there is an antigen-driven unidirectional transfer of miRNA from the T cell to the APC (142). The delivered RNA is functional, and can lead to translation of new proteins in the recipient cell, and/or to regulation of gene expression by miRNA (142).

Summing up, EVs can elicit antigen-specific immune responses by different mechanisms, being able to spread antigens or MHC-peptide complexes, or to directly interact with memory T cells. The intensity and result of these responses depend on the maturation state of the DCs that capture EVs and on the set of molecules carried by EVs.

### Tetraspanins in antigen presentation

Tetraspanins organized in TEMs on the surface of immune cells play an important role in antigen presentation. They are able to interact and recruit different molecules to TEMs forming molecular complexes involved in IS formation (144, 145). Several studies evidence the importance of tetraspanin CD81 in the assembly of a functional immunological synapse. CD81 is present in the central supramolecular activation complex (c-SMAC) (146) and regulates the maturation of the IS on the T cell via its interaction with CD3 and ICAM-1 (147), ensuring a proper and full activation of the T cell. In addition, CD81 associates with CD4 and CD8 playing a role in co-stimulatory signals (148, 149). Mice deficient for CD81 present a delayed humoral response with impaired T and B cell activation (150, 151). On the T cell, CD9 and CD151 also participate in the formation of the IS (152), in this case, by regulating the function of beta1 integrins at the cell–cell contact.

In APCs, tetraspanins CD37, CD53, CD9, CD81, and CD82 have been documented to interact with MHC molecules (153). Tetraspanin–MHC interactions and the recruitment of MHC–peptide complexes in tetraspanin microdomains have been suggested to promote the formation of MHC-II multimers and enhanced antigen presentation (145, 154, 155). CD9 was suggested to be responsible for the higher efficiency of DCs at stimulating naïve T cells compared to other APCs, by forming specialized clusters with MHC-II (155). The contribution of tetraspanins in the organization of MHC clusters has been also reported for tetraspanin CD37 (156). DCs deficient for the expression of either CD37 or CD151 tetraspanins have a hyper-stimulatory effect in T cells but the molecular mechanism differs for each one of these tetraspanins. CD37 plays a role in clustering of MHC and is involved in peptide/MHC presentation, maybe by regulating the interaction of MHC with other tetraspanins that promote MHC clustering such as CD9 and CD82. In contrast, CD151 mediates a co-stimulatory activity (156).

It is therefore feasible that tetraspanins regulate the multimeric state of MHC complexes on the EV membrane contributing to the capacity of EVs to present antigen and activate naïve CD4^+^ T cells (Figure 1D). Thus, tetraspanins, besides regulating the expression and sorting of MHC to EVs, may also regulate the proper degree of clustering in the surface of theses EVs necessary for eliciting an immune response.

With the progressive knowledge in this area, many efforts are focused on the development of proper immunotherapeutic treatments. Cell-free vaccines are being created on the basis of DC-derived exosomes, which have the ability to activate CD4^+^, CD8^+^ T cells, and NK cells. In cancer, tumor-derived exosomes have been used to carry tumor-antigens to induce the anti-tumor responses that would result in tumor cell death. Modulating the antigen-presentation capacity of exosomes by tetraspanin-targeting may have important consequences in this type of promising immunotherapies.

## Concluding Remarks

Further experimental evidences are required to fully define the functional role of tetraspanins in the different aspects of EV biology. However, in light of their potential role in all the processes ranging from EV biogenesis to uptake, tetraspanin-targeting strategies may have a great therapeutic value. Different tetraspanin-targeting strategies have been already described in the literature, such as specific blocking antibodies, synthetic soluble peptides comprising the LEL sequence (14), or cytopermeable peptides with the cytoplasmic region of tetraspanins (35). All these experimental approaches could be envisioned as promising targets for the use of EV in the clinical practice.

## Conflict of Interest Statement

The authors declare that the research was conducted in the absence of any commercial or financial relationships that could be construed as a potential conflict of interest.
